# Composition of Lignin-to-Liquid Solvolysis Oils from Lignin Extracted in a Semi-Continuous Organosolv Process

**DOI:** 10.3390/ijms18010225

**Published:** 2017-01-23

**Authors:** Camilla Løhre, Hilde Vik Halleraker, Tanja Barth

**Affiliations:** Department of Chemistry, University of Bergen, Allégt. 41, 5007 Bergen, Norway; hilde.v.halleraker@uib.no (H.V.H.); tanja.barth@uib.no (T.B.)

**Keywords:** organosolv, lignin, thermochemical conversion, lignin-to-liquid, quantification, phenols

## Abstract

The interest and on-going research on utilisation of lignin as feedstock for production of renewable and sustainable aromatics is expanding and shows great potential. This study investigates the applicability of semi-continuously organosolv extracted lignin in Lignin-to-Liquid (LtL) solvolysis, using formic acid as hydrogen donor and water as solvent under high temperature–high pressure (HTHP) conditions. The high purity of the organosolv lignin provides high conversion yields at up to 94% based on lignin mass input. The formic acid input is a dominating parameter in lignin conversion. Carbon balance calculations of LtL-solvolysis experiments also indicate that formic acid can give a net carbon contribution to the bio-oils, in addition to its property as hydrogenation agent. Compound specific quantification of the ten most abundant components in the LtL-oils describe up to 10% of the bio-oil composition, and reaction temperature is shown to be the dominating parameter for the structures present. The structural and quantitative results from this study identify components of considerable value in the LtL-oil, and support the position of this oil as a potentially important source of building blocks for the chemical and pharmaceutical industry.

## 1. Introduction

One of the keys to a successful biorefinery is maximising valorisation of input feedstock. Lignocellulosic biomass (LCBM), such as wood and grasses, is a promising feedstock in a biorefinery concept due to its natural abundance and for lack of competition with the food industry [1]. LCBM’s major constituents are cellulose, hemicellulose and lignin, with varying ratio between the three fractions depending on plant species and from what part of the plant the biomass is collected [2]. Lignin is the only large-volume renewable feedstock that is composed of aromatics. It is an amorphous cross-linked polymer that gives structural integrity to plants, making up 25% to 35% of woody biomass [3]. Great advances have been achieved in the research aimed at using cellulose as a raw material for bio-based fuels, functional polymers and materials [4], while further development of new processes that generate value-added products from lignin is still needed [5]. Being the most abundant source of renewable phenolic groups, lignin has been seen as a potential replacement of phenol in different types of dispersing agents or thermoset resins, such as phenol–formaldehyde resins. Lignin can also be used in low-cost carbon fibres, engineered plastics and thermoplastic elastomers, polymeric foams, fungible fuels and commodity chemicals. Lignin has a good capacity to adsorb heavy metals ions and has thus been studied as a potential low cost adsorbent for wastewater purification [5,6,7,8].

Lignin has great potential as source for fuel and value-added chemicals [5], and research has been done with the aim of converting lignin to bio-oil that can be utilized as fuel. In addition, a wide range of value-added products, especially phenols, can be derived from lignin using different conversion processes [9]. However, for fuel products one of the challenges with the bio-oil products are their oxygen rich and acidic characters, which result in corrosive properties and a low heating value [10,11]. Lignin conversion into bio-oil using Lignin-to-Liquid-solvolysis (LtL-solvolysis) addresses these challenges. LtL-solvolysis involves hydrothermal conversion of lignin with an in-situ hydrogen donor (formic acid) and a suitable solvent (water or ethanol). LtL-oil is more oxygen rich than crude petroleum, although lignin undergoes substantial deoxygenation during LtL-solvolysis which significantly reduces the oxygen content of the resulting bio-oils [12,13].

Qualitative characterization of the LtL-oil has previously been performed, showing varying composition depending on feedstock type and pretreatment method for lignin isolation [14]. Mechanistic studies have been published [15] and the presence of catalysts also seem to affect the chemical composition of LtL-oils [16]. By quantifying the compounds that comprise the LtL-oils it is possible to evaluate their potential as feedstock for value-added chemicals. The majority of the oil products from the LtL-process are polar phenolic compounds with poor gas chromatographic properties, which makes them hard to quantify by use of gas chromatography. However, if the polar compounds are silylated, using, e.g., bis(trimethylsilyl)trifluoroacetamide (BSTFA), the elution properties are enhanced, the peak symmetry is improved and quantification of the different phenolic compounds is more precise [17,18].

As LCBM comprises considerable quantities of cellulose and hemicellulose in addition to lignin, it is necessary to fractionate the LCBM to isolate the lignin before it can be processed further by LtL-solvolysis. Organosolv fractionation is a fractionation process which aims at removing the lignin biopolymer from the remainder of the biomass by extracting it from a lignocellulosic feedstocks with an organic solvent or an organic/aqueous solution, resulting in a sulphur-free, high purity and low molecular weight purified lignin fraction [19]. In this work, a mixture of ~90% Norway spruce (*Picea abies*) and limited amounts of Pine (*Pinus sylvestris*) was utilised as feedstock. These species were selected due to their high abundance in Norway. Norwegian forests are expanding due to increased temperatures, increased atmospheric CO_2_ levels and nitrogen rich rainfall [20]. Spruce accounts for nearly 50% of all timber by volume in Norway, birch accounts for the numerical nationwide majority of 4 billion trees, followed by spruce on a second place with 3.1 billion trees [21].

**Figure 1:**
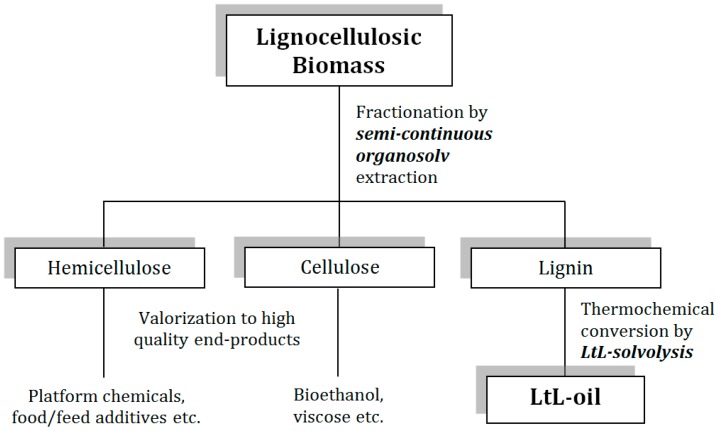
Conceptual sketch including fractionation of lignocellulosic biomass (LCBM) and thermochemical conversion of lignin by LtL-solvolysis.

For efficient use of LCBM as a renewable feedstock for fuels and chemicals, all fractions must be utilised for production of value-added products. Since lignin is the least utilised feedstock fraction, developing processes that efficiently convert this fraction to products are central for development of forestry based biorefineries. The combined processes of lignin extraction and LtL conversion could be very suitable for use in a biorefinery-type approach (see Figure 1).

The aim of this paper is to study the applicability of lignin produced by a *semi-continuous* organosolv fractionation of LCBM for the production of LtL-oil. The lignin is obtained by organosolv extraction, as described in Løhre et al. [22]. The purified lignin fraction is hydrothermally converted by LtL-solvolysis using formic acid (FA) and water.

The effects of LtL-solvolysis reaction conditions on quantitative product yields and quantitative bio-oil composition are studied by applying experimental designs and multivariate data analysis in addition to mass recovery- and carbon mass balance calculations. Conversion temperature and amount of FA are used as variable experimental factors. The LtL-oils are derivatised and analysed by gas chromatography-mass spectrometry/gas chromatography-flame ionisation detection (GC-MS/GC-FID) to investigate compositional differences influenced by reaction conditions.

## 2. Results

### 2.1. Organosolv Fractionation and Product Purity

Three organosolv fractionation experiments were performed under identical reaction conditions to produce lignin for LtL-solvolysis. Average product yields after ended extractions are shown in Table 1.

The average yield of extracted lignin is 83.0% of the total lignin content shown in Table 1. Analysis of the fibre residue showed a residual lignin content of 6.1 wt %. The precipitated lignin fraction was determined to be of 94.9% purity. More detailed information and discussion regarding fractionation process, optimization and organosolv extraction reproducibility is available in previously published work [22].

Precipitated lignin fractions from organosolv fractionation experiments were combined before further use in LtL-solvolysis.

### 2.2. LtL-Solvolysis

An overview of the experimental results from LtL-solvolysis is given in Table 2. A fixed amount of 0.50 g lignin and 4.0 mL distilled water was added to the reactor in each experiment in addition to the varying amount of formic acid. The reaction time was kept constant at two hours. An extended overview of input material, reaction conditions and product output can be found in Supplementary Material (Table S1).

LtL-oil yields and yields of solids/char are calculated as mass fraction of lignin input. Experiment WO.1.2 provides the highest yield of bio-oil (94.2%). This experiment was conducted with high level of hydrogen donor (1.00 mL FA) and low level of temperature (320 °C). Experiment WO.3.1 provides the lowest yield of bio-oil (69.2%). This experiment was conducted with low level of hydrogen donor (0.50 mL FA) and high level for temperature (360 °C).

Four out of six experiments results in an oil yield above 88%. This documents a high conversion ratio, which is confirmed by both the low amount of solid residue and the overall high mass recovery of the experiments. The replicate experiments show good reproducibility for all quantitative parameters; gas phase, aqueous phase, yield of LtL-oil and yield of solid phase.

The experiments are performed in batch at small laboratory scale, thus resulting in an inevitable loss of product during work-up. With this in mind, the mass recoveries obtained are considered good.

### 2.3. Quantification of Individual Components

**Figure 2:**
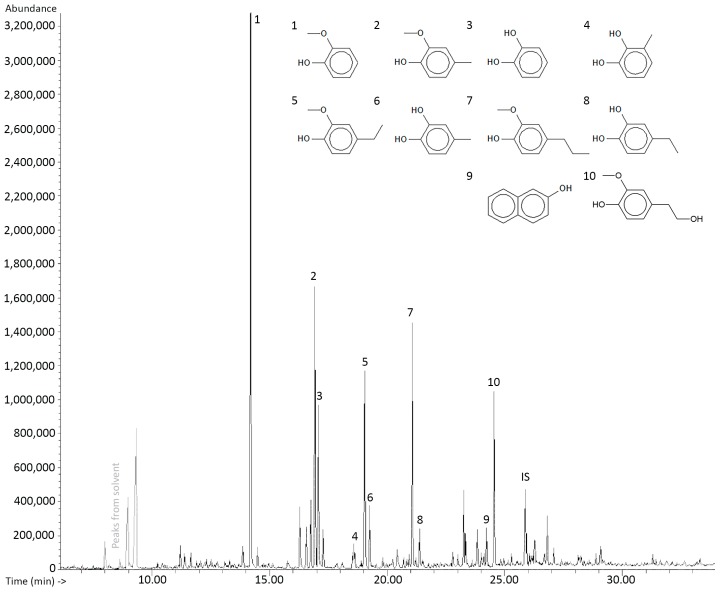
GC-MS chromatogram of experiment WO.3.2 representing a typical compositional distribution. Hexadecane was used as internal standard (IS).

GC-MS library search gave a tentative identification of the compounds present in the bio-oils. The ten compounds with the average highest chromatographic peak areas were selected and used in quantification by GC-FID. The identification of each compound was confirmed by identical retention times as the standards and by identical mass spectra. Figure 2 depicts a GC-MS chromatogram of experiment WO.3.2 showing a representative structural distribution for the LtL-oils. In total, 4.1–9.5 wt % of the bio-oils were accounted for by the ten compounds selected for this study. Table 3 shows all results from the quantitative analysis. Replicate analyses are denoted by the letters “a” and “b”.

Experiments conducted at 320 °C (WO.1.1 and WO.1.2) and 340 °C (WO.2.1 and WO.2.2) produce similar bio-oils. Experiments conducted at 360 °C (WO.3.1 and WO.3.2) produce more guaiacol, catechol, 3-methylcatechol, 4-ethylcatechol, and 2-naphthol than the lower temperature experiments. The amount of 4-methylguaiacol and 4-ethylguaiacol, on the other hand, has a maximum in the centre point experiments and conducted at 340 °C (WO.2.1 and WO.2.2). 

#### Sample Shelf Life of Silylated Bio-Oil

Li et al. reported that silylated samples with excess BSTFA have reduced shelf lives as BSTFA can react with the septum in the vial cap via the syringe injection [17]. In order to investigate the sample’s shelf life, two replicate samples from experiment WO.1.1 were run every week for a month. The sample that was analysed without workup and the water treated sample should ideally produce identical results with regards to quantification of compounds. Inspection of the chromatograms from Week 1 show that the sample treated with water in the aim of hydrolysing excess BSTFA has a considerably lower concentration than the reference sample, indicating that some of the sample is lost in the process of drying or during filtration. The untreated sample produced identical chromatograms throughout the testing period of one month, which means that peak responses were identical and no new peaks appeared. As for the sample treated with water, the responses continued to decline from week to week. No new peaks were detected in the chromatogram, however sample deposits on the wall of the vial were observed.

A standard solution of isoeugenol (mix of *cis* and *trans*) was also analysed by GC-MS one week after the original run on GC-FID without replacing the cap. The GC-MS chromatogram showed distinctive peaks of silicon oxides and can be found in Supplementary Material (Figure S1).

### 2.4. Elemental Analysis

**Figure 3:**
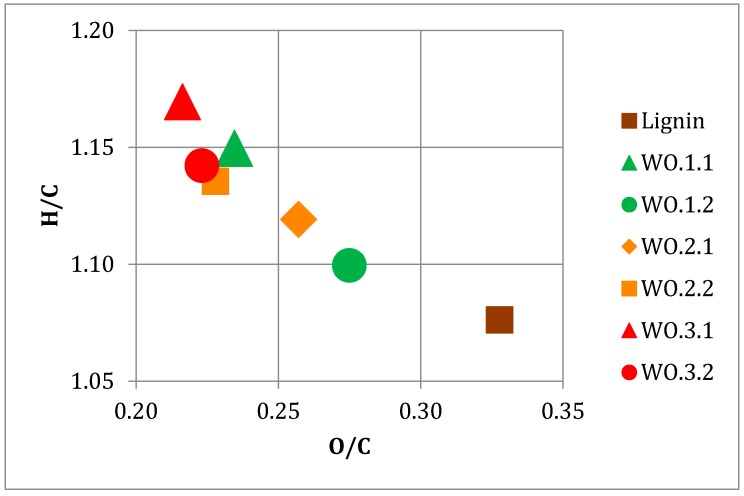
Van Krevelen plot showing H/C ratio and O/C ratio of LtL-oils and lignin.

Figure 3 displays a van Krevelen plot of H/C ratio and O/C ratio of LtL-oils and lignin. The starting biomass sample was also analysed for elemental composition, but is located outside the plot area with O/C and H/C values of 0.74 and 1.61, respectively. Hydrodeoxygenation is clear for all six bio-oils, with all having a lower O/C ratio and higher H/C ratio than the lignin material used as starting material. Experiments WO.1.1 and WO.3.1 show the highest H/C ratios, with both experiments conducted at low value of FA (0.50 mL). This same trend, by negative correlation between H/C and FA (mL), was discovered in the interpretation of the experimental design in Section 3.1.1, thus verifying the observation. Experiments WO.3.1 and WO.3.2 both show the lowest O/C ratio in the plot. A negative correlation between *T* (°C) and O/C ratio was also observed in Section 3.1.1, thus verifying that high reaction temperatures give low O/C ratios (high degree of deoxygenation at high temperatures). Both experiments with high values of FA (1.0 mL), WO.1.2 and WO.3.2, show a slight increase in O/C ratio compared to the experiments with low values of FA (0.5 mL), WO.1.1 and WO.3.1, at the same temperature. High yield experiment WO.1.2 shows the highest O/C ratio. A higher O/C ratio, resulting from a lower degree of deoxygenation, provides additional mass to the bio-oil product, thus a higher yield of bio-oil, by mass, as seen in Table 2.

Replicate centre points display a similar placement in the plot regarding the H/C ratio, but display a difference in O/C ratio of 0.03. Due to the small amount of residue produced, this is within the measurement uncertainty of the oxygen content determination (determined by difference).

### 2.5. Carbon Balance

Table 4 displays carbon balance calculations from all LtL-solvolysis experiments. Mass percentages of carbon in lignin, bio-oils and char/solid product were determined using elemental analysis. The balance includes input of carbon in the form of lignin, and output of carbon in the form of bio-oil product, char/solid product and the aqueous phase. Calculations show a balance between 118% and 140% for all experiments, which is unexpected. This can, to some degree, be explained by residual amounts of the extraction solvents in the aqueous phase, as will be discussed in Section 3.2, but even excluding all aqueous contributions the carbon recovery is higher than 100%. ^1^H-spectra and ^1^H-^1^H-COSY-spectra of the aqueous phase from experiment WO.2.1 can be found in Supplementary Material (Figures S3–S6), and show that the aqueous organic compounds comprise both solvent residues and lignin derived compounds.

## 3. Discussion

Both experiments with low levels of formic acid provide the lowest yields of bio-oil (WO.1.1 and WO.3.1). Experiments with a high level of formic acid (WO.1.2 and WO.3.2) do not provide the highest yield of bio-oil, as seen by centre point experiments (WO.2.1 and WO.2.2) both providing a higher yield of bio-oil than experiment WO.3.2. This observation suggests the amount of formic acid only needs to reach a sufficient level to induce depolymerisation and hydrogenation of the lignin polymer.

### 3.1. Experimental Design and Multivariate Data Analysis

#### 3.1.1. LtL-Solvolysis

**Figure 4:**
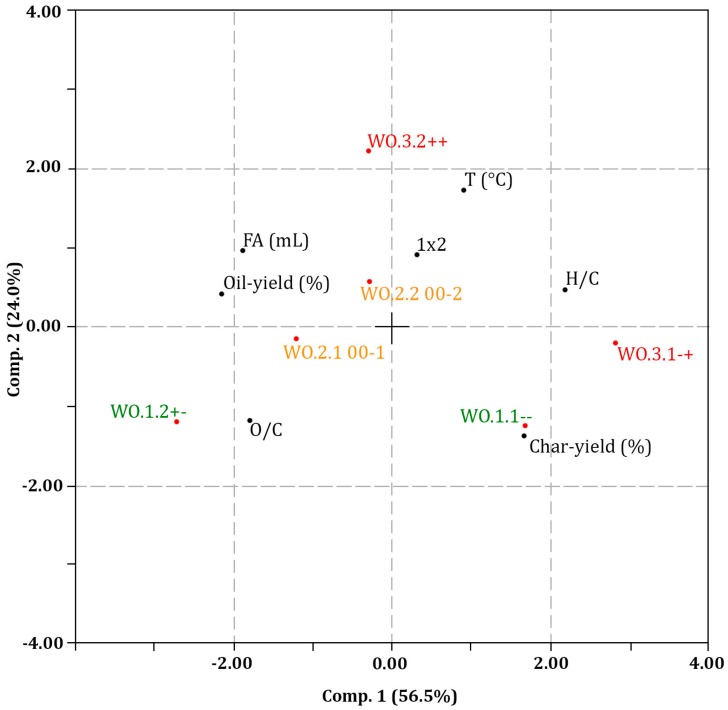
Biplot from principal component analysis (PCA) of the dataset consisting of experimental variables (including the cross term between FA and temperature 1 × 2) and major response groups. Colour coding corresponds to the Van Krevelen plot in Figure 3. High (+) and low (-) values, together with intermediate centre points (0), for the variables V1 (FA loading) and V2 (reaction temperature) are included in the plot.

The experimental variables and responses from LtL-solvolysis were subjected to principal component analysis (PCA). Replicate centre points are included in the plot (WO.2.1 and WO.2.2). Figure 4 displays a biplot of the variables and major response groups. The data are standardised, meaning that all values for each variable are scaled by division with the standard deviation, giving an equal variance from −1 to +1 for each variable to remove effects of the different numerical ranges.

The following clear relationships are shown in the plot: The oil yield (%) is negatively correlated with H/C ratio; illustrating a high yield bio-oil corresponds to a lower H/C ratio. The oil yield (%) is negatively correlated with char yield (%), illustrating that a high yield of bio-oil naturally reduces the capacity for production, and hence the yield, of solid residue/char. Correspondingly, a positive correlation between FA (mL) and oil yield (%), and a negative correlation between FA (mL) and char yield (%) is clear, illustrating a high/sufficient amount of formic acid increases the oil yield (%) and reduces the char yield (%). FA (mL) is also positively correlated with O/C ratio on the first component, illustrating an increase in O/C ratio with an increased amount of formic acid in the LtL-solvolysis reaction. The temperature (°C) shows a clear negative correlation with O/C ratio and positive correlation with H/C ratio, illustrating high reaction temperatures giving a more efficient hydrodeoxygenation. A subsequently expected negative correlation is also observed between H/C ratio and O/C ratio, verifying the hydrodeoxygenation process. A trend of decreasing O/C ratio with increased temperature, and increasing O/C ratio with increased addition of FA (mL) is also seen in the van Krevelen diagram in Figure 3.

A positive correlation could be expected between H/C ratio and FA (mL) since FA is thought to provide the active hydrogen for hydrogenation, but this is not seen in the plot. On the contrary, there is a negative correlation between H/C ratio and FA (mL). This may be explained by considering the reaction of the methoxy groups: If a methoxy substituent in a methoxy-phenol is hydrogenated, it will provide phenol and methanol. This would in principle imply an increased overall H/C ratio due to hydrogenation. However, the methanol would be found in the aqueous phase and would be removed at an early stage during workup (and/or some may occur in the organic phase and be evaporated off during solvent removal at the end of workup) [15]. Thus, an H/C ratio of 8/7 (methoxy-phenol) results in an H/C ratio of 6/6 (phenol) for the bio-oil component, hence the H/C ratio decreases upon hydrogenation. Spruce and pine are softwoods and softwood lignins are known to contain 90%–95% of the coniferyl alcohol monomer with only one methoxy substituent on the phenylpropane unit, and 5%–10% of the sinapyl alcohol monomer containing two methoxy substituents on the phenylpropane unit [23]. Though no phenol was discovered by GC-MS or GC-FID analysis using standard solutions, various methoxy-phenols and catechols were identified and can be assumed to follow this reaction pathway (see Table 3). The effect would be larger at higher conversion, since reaction duration of only two hours would be insufficient to induce complete hydrogenation of all constituents during depolymerisation.

The cross-term 1 × 2, representing both T (°C) and FA (mL), explains the combined effect of the two variables have on a given response. The cross-term can be seen as expressing the overall severity of the conversion conditions. The cross-term shows a negative correlation with the O/C ratio and minor positive correlation with the H/C ratio. This confirms the hydrodeoxygenation-type conversion as the main reaction overall.

#### 3.1.2. Quantitative Analysis

##### Sample Shelf Life of Silylated Bio-Oil

No new peaks were observed in the chromatograms after multiple GC-FID runs and the BSTFA was not given the opportunity to react with the septum as the caps were replaced immediately after each analysis. The sample treated with water displays decreasing responses in the chromatograms. Li et al. claims that use of propanol to treat excess of BSTFA will lead silylated phenolic compounds to be rapidly transformed back to their underivatized form [17]. This should not be the case when water is used, as all the excess water is also removed with sodium sulphate. The sample shelf life of silylated bio-oil was hence found to be at least one month.

##### Quantitative Analysis

**Figure 5:**
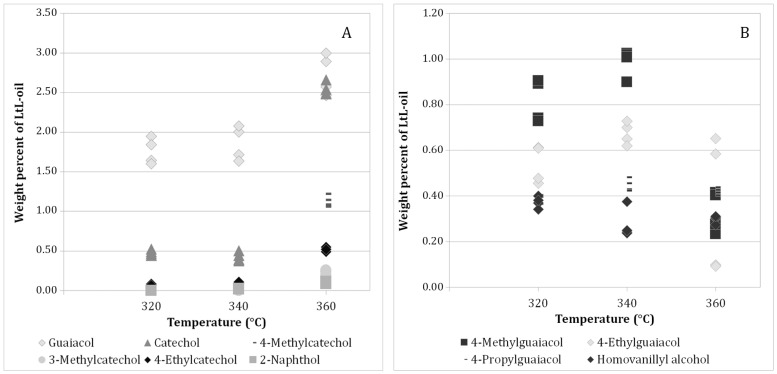
(**A**) Yields of single compounds plotted against reaction temperature for compounds displaying a positive correlation between temperature and yields. (**B**) Yields of single compounds plotted against reaction temperature displaying negative and no correlation between temperature and compound yields.

The experiments form two distinguishable groups with regards to the chemical composition. The experiments performed at 320 °C and 340 °C exhibit similarities, whereas the experiments conducted at 360 °C stand out. These results can be seen both in Table 3 and in Figure 5. A biplot of the principal component analysis of the multivariate data handling can be found in Supplementary Material (Figure S2) and supports this observation. The amount of formic acid used in each experiment is seen to have a less prominent role than temperature in regards to the presence of the different compounds in the bio oil.

The compounds found to be temperature dependent (positively correlated) are guaiacol, catechol, 4-methylcatechol, 3-methylcatechol, 4-ethylcatechol and 2-naphthol. 3-Methylcatechol is not produced at 320 °C or 340 °C, but is produced in small amounts at 360 °C. 2-Naphthol is absent from the oils produced at 320 °C, but is produced in very small quantities in the other experiments.

All compounds that are strongly correlated to temperature are practically unaffected by the amount of formic acid. The amount of hydrogen donor (mL FA) during LtL-solvolysis is negatively correlated to 4-ethylguaiacol, 4-propylguaiacol, homovanillyl alcohol, and to some extent guaiacol and 4-methylguaiacol.

In the biplot in Figure 4, there is a clear negative correlation between temperature (°C) and O/C ratio. In Figure 5, it is clear that guaiacol, catechol, 3-methylcatechol, 4-methylcatechol, 4-ethylcatechol and 2-naphthol are positively correlated with temperature (°C). These six compounds have O/C ratios of 0.29, 0.33, 0.29, 0.29, 0.25 and 0.10 respectively. No clear correlation is seen between O/C ratios in the identified compounds and increased reaction temperature, so this correlation must be explained by the unidentified fraction of the LtL-oils. The quantified compounds together with the internal standard make up 35%–53% of the total peak area in the chromatograms obtained by GC-MS of underivatised bio-oil, indicating that there is a substantial part of the oil that is not sufficiently volatile for GC-MS analysis and thus impossible to analyse by this method. At harsher conditions, meaning at higher temperatures and longer reaction times, or by the use of catalysts, one could expect to find more of the compounds suitable for GC-MS analysis and consequently be able to identify and quantify a larger fraction of the bio-oil. This is supported by the findings of Kalogiannis et al. where a larger fraction of catechols are observed at higher temperatures and with the use of catalysts [24]. Using a semi quantitative method, Wang et al. reported a declining fraction of guaiacols at more severe conditions [25]. This observation is also supported by Kalogiannis et al. [24], and the same trend can be seen in our data where the amount of substituted guaiacols decline at the highest reaction temperature. The amount of non-substituted guaiacol, however, increases with higher temperatures in the range applied in our experiments. It is reasonable to assume that at harsher experimental conditions, guaiacol will be transformed into catechol or phenol [15,26]. It is worth noting that the oil yields in the cited studies [24,25] are 18%–35% and 30%–42%, respectively, and our work has given oil yields up to 94% based on lignin input. 

Quantification results from this study indicate the possibility of tuning experimental LtL-solvolysis conditions towards preferable product composition for utilisation in the chemical industry. Guaiacol, which is found in large quantities in all of the analysed LtL-oils, is an agent thought to have disinfectant properties and is used as an expectorant in the pharmaceutical industry. Guaiacol can also be found as a flavour in foods as roasted coffee and contributes to the taste of smoked meat [27,28]. 4-Methylguaiacol, 4-ethylguaiacol and 4-propylguaiacol are all phenol derivatives that can be used as flavouring agents [29]. Catechol is commonly used as a photographic developer, as an intermediate for antioxidants in rubber and lubricating oils, in polymerisation inhibitors, in fur dyes and leather tanning, as well as in pharmaceuticals [30,31]. 2-Naphthol is a widely used intermediate for the production of dyes [32]. Homovanillyl alcohol (HVA) is found as a key component of Queen mandibular pheromone (QMP), produced by honey bee queens, and used to regulate the behaviour and physiology of their nest mates. QMP has shown to block aversive learning in young worker bees, an effect that can be mimicked by treating bees with HVA [33]. This supports the search for other bioactive compounds in the LtL-oils.

The compound structures quantified by GC-FID show great similarities in the distributions of the phenolic monomers in all the samples. In future work, such detailed quantification could be reduced to quantifying series of homologs or otherwise similar compounds. Separation of LtL-oil into desired homologs, followed by further separation and/or quantification, could increase the LtL-application potential even further. The identified structures and quantitative results from this study thus suggest a potential for recovering high value components from the LtL-oil, and supports the potential for using it as a source of building blocks for the chemical and pharmaceutical industry.

### 3.2. Reproducibility, Mass Recovery and Carbon Balance

In terms of yields of the desired bio-oil the reproducibility is very good. The two replicate experiments WO.2.1 and WO.2.2 produce 89.3% and 89.4% bio-oil, respectively, based on the mass of lignin input. The amount of solids produced is also low with yields of 7.5% and 7.0%, respectively. Due to limited amounts of feedstock, small scale experiments were performed and minimal variations during workup procedures can affect the quantitative results significantly. However, workup procedures seem to be consistent.

Mass recovery from semi-continuous organosolv fractionation was determined at 99% and is considered good. Mass recovery in all LtL-solvolysis experiments exceeds 96% and is considered acceptable on the basis of small amounts of feedstock and product. Thus, the combined processing results in a very high utilisation of the lignin in the starting material.

High levels of dissolved organic carbon are detected in the aqueous phases as seen in Table 4. ^1^H-NMR analysis of the aqueous phases from experiment WO.2.1 shows a presence of both the extraction solvents, EtAc and THF, in addition to significant quantities of methanol, ethanol and considerable amounts of components such as aromatics and carboxylic acids (Figures S3–S6). The solvents, EtAc and THF, will give a false contribution of carbon in the carbon balance calculations as they are not included in the calculations based on carbon input. The latter compounds discovered in the aqueous phase originate from the lignin and thus contribute to the mass and element balance relative to lignin input. The production of methanol from lignin during the conversion is already discussed in Section 3.1.1. Other components such as carboxylic acids are thought to potentially originate from formic acid. Previous, unpublished, work indicates a formation of short-chained aliphatic mono-functional acids (C_2_ >> C_3_ > C_4_ > C_5_ > C_6_ > C_7_ > C_8_ > C_n_) during pyrolysis of formic acid in water with goethite mineral (FeOOH) as support [34].

By eliminating the aqueous phase from the calculations, carbon balances over 100% are still detected. This also indicates a potential carbon contribution from formic acid to the bio-oil itself. A net carbon contribution from formic acid could also affect the H/C and O/C ratio of the bio-oils. LtL-experiments using ^13^C-labeled formic acid are considered in this approach and will be investigated further, in addition to quantitative correlations between formic acid products in the aqueous phase after LtL-solvolysis. A mechanism that incorporates formulation of lignin functional groups by formic acid in catalysed lignin depolymerisation has recently been published [35], and similar mechanisms can be envisioned in non-catalysed LtL as well.

## 4. Materials and Methods

All solvents in the experimental work were purchased from Sigma Aldrich (St. Louis, MO, USA) and used without any further purification. All standard compounds are commercially available. 

### 4.1. Organosolv Fractionation and Product Purity

The biomass used in the experimental setups consisted of industrially obtained wood shavings from softwood, comprising ~90% Norway spruce (*Picea abies*) and limited amounts of Pine (*Pinus sylvestris*). It was kindly supplied by Weyland AS in Bergen, Norway, produced from locally grown wood in the district of Hardanger, Norway, and processed at Granvin Bruk in Hardanger. Detailed biomass characteristics are available in Løhre et al. [22]. The feedstock was used as received.

Three organosolv fractionation experiments were conducted under identical reaction conditions to produce lignin for LtL-solvolysis. The method applied for organosolv experiments is described briefly. More detailed information regarding equipment, fractionation process, optimization and product purity is available in Løhre et al. [22].

Wood shavings (>500 μm) were loaded into a custom made 250 mL high pressure stainless steel column. A solution of 63 wt % ethanol in distilled water, containing sulfuric acid as catalyst (6.0 mM), was used as solvent for the organosolv fractionation. Solubilised and dissolved lignin was pumped out of the column in a continuous stream and collected in a beaker. Solvent flow was kept constant at 1.500 mL·min^−1^, fractionation temperature was kept constant at 175 °C, system pressure was held at 20 bars to ensure solvents being kept in liquid state during extraction and extraction time was kept for 10 h.

After the extraction period was complete, an amount of solvent equivalent to the total system volume (255 mL) was pumped through to wash the solid residue when the system reached ambient temperature. The washing solution was combined with the organosolv liquor. The solid residue was dried at ambient temperature to constant mass. The organosolv liquor was diluted with distilled water in a ratio of 1:3 *v*·*v*^−1^ (organosolv liquor:H_2_O), cooled to 4 °C and left for precipitation. The precipitated lignin was filtered off and dried at ambient temperature to constant mass. The ethanol and water solvent was removed from the aqueous phase on a rotary evaporator at a temperature of 40 °C and a pressure of 175 mbar and 65 mbar to recover solvent fractions and to determine mass recovery of hemicellulose and/or water soluble residuals in the aqueous phase.

Product purity of selected samples of organosolv fibre residue and lignin precipitate has previously been reported by employing hydrolysis according to protocol NREL/TP-510-42618 [36]. Acid insoluble lignin (Klason lignin) was determined gravimetrically and acid soluble lignin was determined spectrophotometrically according to protocol TAPPI UM 250 [37].

### 4.2. Experimental Design and Reaction Conditions for LtL-Solvolysis

LtL-solvolysis experiments were set up as an experimental design to screen the effect of selected experimental factors on the amount and composition of the products. The range of reaction conditions used was based on optimal conditions found in previous work [14,16,38]. Experimental variables were (V1) loading of hydrogen donor (mL FA) and (V2) reaction temperature (°C). Reaction duration (2 h), lignin loading (0.5 g) and loading of distilled water (4.0 mL) were kept constant. Table 5 gives an overview of the experimental parameters. High (+) and low (-) values, together with intermediate centre points (0), for the variables V1 (FA loading) and V2 (reaction temperature) were selected for use in the design.

Both quantitative yields and bio-oil compositional quantitative results from the design were interpreted using principal component analysis (PCA) and Sirius 8.1 software. A biplot of a PCA reveals correlations between descriptors/loadings and their potential association with the properties of an object. The first component (Comp. 1) is found by linear combination of the original data explaining the maximum of its variance. The second component (Comp. 2) describes the maximum of the variance which cannot be described by the first component. If the loadings are projected close to each other with respect to the origin they are positively correlated, and if they are projected opposite (180°) to each other with respect to the origin they are negatively correlated. Descriptors which have a strong influence on the model will be projected far from the origin, and descriptors with negligible or minor influence will appear close to the origin in the loading plot/biplot [39].

### 4.3. LtL-Solvolysis

Precipitated lignin from organosolv fractionation experiments was combined before further use. Lignin, distilled water and formic acid were added to a 25 mL high pressure Parr reactor from the 4740-series without stirring and placed in a preheated Carbolite Laboratory High Temperature oven. After completed reaction time (2 h), the reactor was removed from the oven and cooled to ambient temperature. The resulting products after solvolysis included a gas phase, a liquid phase and a small amount of solid phase. The amount of gaseous product was determined by weighing the reactor before and after venting the gases. Gas composition analysis was not performed as part of this study, but relevant data for gas composition is previously published by Oregui Bengoechea et al., showing that the main components of the gas phase are the decomposition products, CO_2_, H_2_ and CO, of formic acid and limited amounts of light alkanes such as methane [16]. The pressure during conversion is primarily determined by the steam pressure at the given temperature with a minor additional contribution from FA decomposition, and is in the range of 200–230 bars as measured in comparable conditions at larger scale.

The liquid product consisted of a single aqueous phase with dispersed organic particles. Dark brown LtL-oil was not present as a separate phase, but adsorbed to the solid residue due to its hydrophobic character. The LtL-oil is miscible in an ethyl acetate (EtAc) and tetrahydrofuran (THF) mixture and is therefore separated from the solid phase using EtAc:THF (9:1 *v*·*v*^−1^) and filtered through a 0.45 μm Puradisc^TM^ 25 NYL filter (GE Healthcare, Amersham, UK). The remaining liquid phase was then extracted with EtAc:THF (9:1 *v*·*v*^−1^) three times and the organic phases were combined and dried over Na_2_SO_4_ (s).

The solvent was removed from the LtL-oil on a rotary evaporator at a temperature of 40 °C and a pressure of 175 mbar until stable mass. This pressure is set as standard conditions for all LtL-oils to ensure the same workup-protocol as previous work.

### 4.4. Total Organic Carbon in Aqueous Phase (TOC)

TOC was conducted as a part of mass recovery calculations. Aqueous phases from all LtL-experiments were diluted with a ratio of 1:2500 (*v*·*v*^−1^) and analysed for organic carbon content on a Vario TOC Cube from Elementar (Mount Laurel, NJ, USA).

### 4.5. Elemental Analysis of LtL-Oil and Solid Residue

LtL-oils and solid residue were analysed by elemental analysis in carbon hydrogen nitrogen and sulphur mode with a VarioEL III from Elementar. The amount of oxygen was calculated by difference.

### 4.6. ^1^H-NMR

An aliquot of the aqueous phase sample was diluted with an equal amount of D_2_O and analysed directly. NMR-spectra were acquired using a Bruker Avance 600-instrument and processed with Bruker TopSpin 3.5 (Billerica, MA, USA). ^1^H-spectra were recorded with 64 transients and water signal suppression using presaturation pulses. Magnitude-mode ^1^H-^1^H COSY spectra were recorded using 16 transients and 512 t1 increments.

### 4.7. Quantification

The LtL-oils produced in this study are complex mixtures of lignin degradation products. Qualitative GC-MS analysis of all LtL-oils was conducted to tentatively identify major components in the oils. The ten compounds with the highest average chromatographic area by GC-MS, relative to an internal standard, were selected and used in quantification by GC-FID. Calibration curves were made using GC-FID for all ten compounds and made by linear regression based on multiple standard solutions of each compound. The ratio between the area of an internal standard and the area of the compound in question were used to construct the calibration curves. No correlation coefficient, *R*^2^, of less than 0.997 was accepted. In order to make calibration curves, stock solutions of the standard compounds were made by measuring 0.04 g of the selected compounds into a vial, and adding 10 mL of a dichloromethane:methanol solution (93:7 *v*·*v*^−1^). Volumes of 20/40/60/80 μL of the stock solutions were transferred to separate vials and diluted with 2.0 mL of pentane and 3.0 mL of an internal standard solution (0.05 mg·mL^−1^ hexadecane in pentane) to make the final quantitative standards.

#### 4.7.1. Silylation

One millilitre of each quantitative standard was added to a GC-vial together with 100 μL pyridine and 100 μL bis(trimethylsilyl)trifluoriacetamide (BSTFA) containing 1% trimethylchlorosilane (TMCS). The vial was then capped and heated to 70 °C for 20 min before cooling to room temperature followed by subsequent cooling to 5 °C before analysing by GC-FID.

The LtL-oil samples were prepared by measuring 20 mg bio-oil into a vial, adding 1.5 mL internal standard solution (0.2 mg·mL^−1^ hexadecane in EtAc:THF (9:1 *v*·*v*^−1^)) and 1.5 mL of EtAc:THF (9:1 *v*·*v*^−1^), thus giving a bio-oil concentration of 6.7 mg·mL^−1^. A volume of 1.0 mL of the bio-oil solution was transferred to a GC-vial and 300 μL pyridine and 300 μL of BSTFA + TMCS were added. The GC-vial was capped and heated to 70 °C for 30 min before cooling to room temperature. A volume of 0.5 mL of silylated bio-oil was transferred to a new GC-vial, diluted with 0.5 mL pentane and cooled to 5 °C overnight before running with GC-FID. Two replicate analyses were done for all six bio-oil samples.

#### 4.7.2. Sample Shelf Life of Silylated Bio-Oils

Two silylated oil samples were prepared. One of the samples was treated with 100 μL of water to hydrolyse excess BSTFA, dried with Na_2_SO_4_ and filtered before running both samples with GC-FID. After each run the cap was replaced with an unused cap.

### 4.8. Gas Chromatography

#### 4.8.1. Gas Chromatography-Mass Spectrometry (GC-MS)

Qualitative GC-MS analysis of the LtL-oils was performed using an Agilent Technologies 7890A GC-system, with auto sampler, coupled with an Agilent Technologies 5977A MSD. A sample volume of 1 μL was injected in splitless mode on a 30 m HP-5ms column with 250 μm ID and thickness of 0.25 μm from Agilent Technologies (Santa Clara, CA, USA). Helium was used as carrier gas with a constant flow of 1 mL/min. The injector temperature and detector temperatures were kept at 280 and 250 °C, respectively, and the following GC oven temperature program was applied: Start temperature: 40 °C; (hold time—5 min); Heating rate 1: 6 °C·min^−1^; (hold time—0 min); Final temperature: 280 °C; Heating rate 2: 40 °C·min^−1^; (hold time—5 min); Final temperature: 300 °C.

The GC-MS inter-phase valve delay was set to 4.60 min and the MS detector operated in positive mode with an ion-source temperature of 254 °C. Compounds were detected using Enhanced MSD ChemStation software F.01.00.1903 from Agilent Technologies (Santa Clara, CA, USA) and tentatively identified using the NIST 2.0 library from Agilent Technologies (Santa Clara, CA, USA)

#### 4.8.2. Gas Chromatography-Flame Ionization Detector (GC-FID)

Silylated LtL-oils were analysed on a Thermo Finnigan Trace GC ultra with auto sampler and equipped with a FID detector (Termo Finnigan, Milan, Italy). A sample volume of 1 μL was injected in splitless mode on a 30 m HP-5ms column with 250 μm ID and thickness of 0.25 μm from Agilent Technologies. Helium was used as a carrier gas with a constant pressure of 100 kPa. Injector temperature and the detector temperature were kept at 250 and 330 °C, respectively, and the following GC oven temperature program was applied: Start temperature: 30 °C; (hold time—5 min); Heating rate: 6 °C·min^−1^; (hold time—5 min); Final temperature: 250 °C.

## 5. Conclusions

The aim of this paper was to study the applicability of lignin from semi-continuous organosolv fractionation in hydrothermal conversion using LtL-solvolysis. The effects of solvolysis reaction conditions on quantitative product yields and quantitative bio-oil composition were studied by applying experimental designs and multivariate analysis in addition to mass recovery and mass balance calculations.

The lignin fraction from semi-continuous organosolv fractionation was found highly applicable for the LtL-solvolysis process. Yields of bio-oil were found as high as 94% of lignin mass input. In the range of conditions covered in this study, the bio-oil yield was found to be positively correlated with the amount of hydrogen donor (formic acid) added to the reaction, and so a theory of sufficient amounts/upper limit of hydrogen donor added in an LtL-solvolysis reaction is proposed.

The reaction temperature (°C) during LtL-solvolysis shows a clear negative correlation with the O/C ratio in the bio-oils; illustrating high reaction temperatures giving a high degree of deoxygenation. Bio-oil quantification of the 10 compounds with highest average area in the GC-MS chromatograms yielded structural identification of ≤9.5% of the total oil (by mass), where reaction temperature was the dominating parameter influencing the structures present, and the effect of formic acid volume was limited.

Mass recovery from semi-continuous organosolv fractionation was determined at 99%, mass recovery from LtL-solvolysis was detected as >96% and total carbon mass balances from LtL-solvolysis were detected as >118%. A plausible explanation of carbon balances exceeding 100% due to contributions from formic acid is proposed, and the role of formic acid in the reaction mechanism will be investigated further using ^13^C-labelled formic acid in LtL-experiments.

## Figures and Tables

**Table 1:** Average results from organosolv extraction.

Wood Shavings (g)	25.6
Ethanol:Water (wt %)	63:37
Temperature (°C)	175
Flow (mL·min^−1^)	1.500
Time (h)	10.0
Sulfuric acid—H_2_SO_4_ (mM)	6.00
Fibre residue (wt % of input biomass)	25.8 (±1.0)
Fibre residue (wt % of sample’s cellulose mass)	68.1 (±2.7)
Residual lignin content in fibre residue (wt %)	6.1 (±0.1)
Lignin yield (wt % of input biomass)	23.9 (±0.8)
Lignin yield (wt % of sample’s lignin mass)	83.0 (±2.9)
Lignin purity (%)	94.9 (±0.2)
Residuals in aqueous phase (wt % of input biomass)	49.1
Sum of fibre residue, extracted lignin and residuals in Aqueous phase (wt % of input wood shavings mass)	99.1

**Table 2:** Yields from LtL-solvolysis.

In/Out	Experiment	WO.1.1	WO.1.2	WO.2.1	WO.2.2	WO.3.1	WO.3.2
In	Formic acid (mL)	0.50	1.00	0.75	0.75	0.50	1.00
	Temperature (°C)	320	320	340	340	360	360
	Total mass input (g)	5.12	5.78	5.45	5.45	5.18	5.76
Out	Gas (% of formic acid input)	98.4	90.2	96.8	96.8	96.8	98.4
	LtL-oil yield (% of lignin input)	77.9	94.2	89.3	89.4	69.2	88.7
	Aqueous-phase (% of solvent input) *	97.3	99.1	97.4	97.1	99.3	96.6
	Solids (% of lignin input)	22.7	9.7	7.5	7.0	19.1	5.7
	Total mass output (g)	5.00	5.70	5.30	5.29	5.07	5.57
	Mass recovery (%)	97.7	97.6	97.2	96.9	97.9	96.8

* All aqueous-phases were measured to pH = 5.0–5.5.

**Table 3:** Mass percentage of selected compounds in bio-oil samples. Replicate analyses are denoted by the letters “a” and “b”.

Compound	O/C Ratio	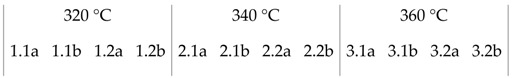
Guaiacol 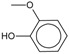	0.29	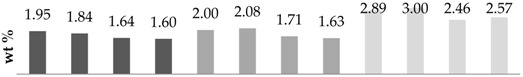
4-Methylguaiacol 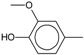	0.25	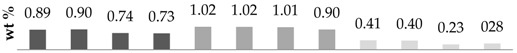
4-Ethylguaiacol 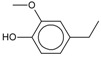	0.22	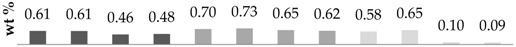
4-Propylguaiacol 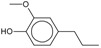	0.20	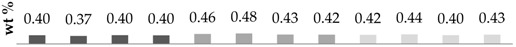
Catechol 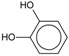	0.33	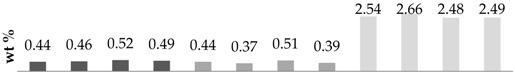
3-Methylcatechol 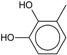	0.29	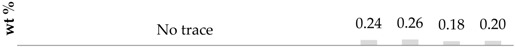
4-Methylcatechol 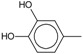	0.29	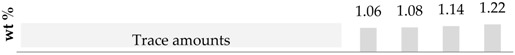
4-Ethylcatechol 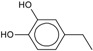	0.25	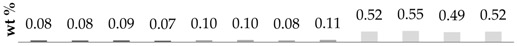
2-Naphthol 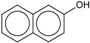	0.10	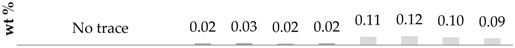
Homovanillyl alcohol 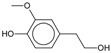	0.33	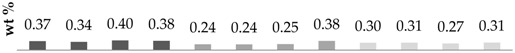
**Total identified (wt %)**		4.74 4.60 4.25 4.14 4.99 5.05 4.66 4.47 9.08 9.47 7.88 8.20

**Table 4:** Carbon balance in LtL-solvolysis.

Experiment	WO.1.1	WO.1.2	WO.2.1	WO.2.2	WO.3.1	WO.3.2
Lignin mass (g)	0.51	0.51	0.50	0.50	0.51	0.51
Carbon content in Lignin (wt %)	65.36					
Carbon added as Lignin (g)	0.33	0.33	0.33	0.33	0.33	0.33
Bio-oil mass (g)	0.40	0.48	0.45	0.45	0.35	0.45
Carbon content in Bio-oil (wt %)	70.66	68.50	69.53	71.19	72.05	71.71
Carbon contribution from Bio-oil (g)	0.28	0.33	0.31	0.32	0.25	0.32
Char mass (g)	0.12	0.05	0.04	0.04	0.10	0.03
Carbon content in Char (wt %)	67.48	47.25 *	51.92	48.06 *	72.62	NA **
Carbon contribution from Char (g)	0.08	0.02	0.02	0.02	0.07	NA **
Carbon mass in aqueous sample (g)	0.10	0.11	0.06	0.06	0.13	0.11
Carbon sum from products (g)	0.46	0.46	0.39	0.39	0.45	0.44
Total Carbon balance (%)	138.86	139.58	118.74	119.93	136.29	>134.2
Carbon balance excluding the aqueous phase (%)	107.67	105.72	100.94	102.52	97.52	>97.3

* Low char yields give high concentrations of ashes and low carbon contents in the recovered solids; ** NA = Insufficient sample amount for determination.

**Table 5:** Experimental details for LtL-solvolysis experiments: V1 (-) = 0.50 mL, V1 (0) = 0.75 mL, V1 (+) = 1.00 mL, V2 (-) = 320 °C, V2 (0) = 340 °C and V2 (+) = 360 °C.

Experiment	WO.1.1 --	WO.1.2 +-	WO.2.1 00-1	WO.2.2 00-2	WO.3.1 -+	WO.3.2 ++
V1	-	+	0	0	-	+
V2	-	-	0	0	+	+

## Supplementary Materials

Supplementary materials can be found at www.mdpi.com/1422-0067/18/1/225/s1.
