# Supplementary material for: Composition of Lignin-to-Liquid Solvolysis Oils from Lignin Extracted in a Semi-Continuous Organosolv Process
Source: Int J Mol Sci. 2017 Jan 23;18(1):225. doi: 10.3390/ijms18010225 (PMC5297854; doi:10.3390/ijms18010225)
Supplement: Supplementary file 1 [file ijms-18-00225-s001.pdf]

# Supplementary Materials: Composition of Lignin-to-Liquid Solvolysis Oils from Lignin Extracted in a Semi-Continuous Organosolv Process

**Camilla Løhre, Hilde Vik Halleraker and Tanja Barth**

**Table S1.** Extended overview of reaction conditions, input material and product output from LtL-solvolysis. High (+) and low (-) values, together with intermediate centre points (0), for the variables V1 (FA loading) and V2 (reaction temperature) are included in the table.

| In/Out | Experiment | WO.1.1 | WO.1.2 | WO.2.1 | WO.2.2 | WO.3.1 | WO.3.2 |
|---|---|---|---|---|---|---|---|
| | V1 V2 | -- | +- | 00-1 | 00-2 | -+ | ++ |
| In | Lignin (g) | 0.51 | 0.51 | 0.50 | 0.50 | 0.51 | 0.51 |
| | Formic Acid (g) | 0.61 | 1.22 | 0.93 | 0.93 | 0.62 | 1.22 |
| | V1 Formic Acid (mL) | 0.50 | 1.00 | 0.76 | 0.76 | 0.51 | 1.00 |
| | V2 Temperature (°C) | 320 | 320 | 340 | 340 | 360 | 360 |
| | Water (g) | 4.00 | 4.05 | 4.02 | 4.02 | 4.05 | 4.03 |
| | Total mass input (g) | 5.12 | 5.78 | 5.45 | 5.45 | 5.18 | 5.76 |
| | Lignin percentage of mass input (%) | 9.9 | 8.8 | 9.2 | 9.2 | 9.8 | 8.8 |
| Out | Gas (g) | 0.60 | 1.10 | 0.90 | 0.90 | 0.60 | 1.20 |
| | LtL-oil (g) | 0.40 | 0.48 | 0.45 | 0.45 | 0.35 | 0.45 |
| | Solids (g) | 0.12 | 0.05 | 0.04 | 0.04 | 0.10 | 0.03 |
| | Aqueous-phase (g) | 3.89 | 4.01 | 3.91 | 3.90 | 4.02 | 3.89 |
| | Total mass output (g) | 5.00 | 5.70 | 5.30 | 5.29 | 5.07 | 5.57 |
| Results | Gas (% of FA input) | 98.4 | 90.2 | 96.8 | 96.8 | 96.8 | 98.4 |
| | Aqueous-phase (% of solvent input) | 97.3 | 99.1 | 97.4 | 97.1 | 99.3 | 96.6 |
| | Solids (% of lignin input) | 22.7 | 9.7 | 7.5 | 7.0 | 19.1 | 5.7 |
| | LtL-oil yield (% of lignin input) | 77.9 | 94.2 | 89.3 | 89.4 | 69.2 | 88.7 |
| | Mass recovery (%) | 97.7 | 97.6 | 97.2 | 96.9 | 97.9 | 96.8 |

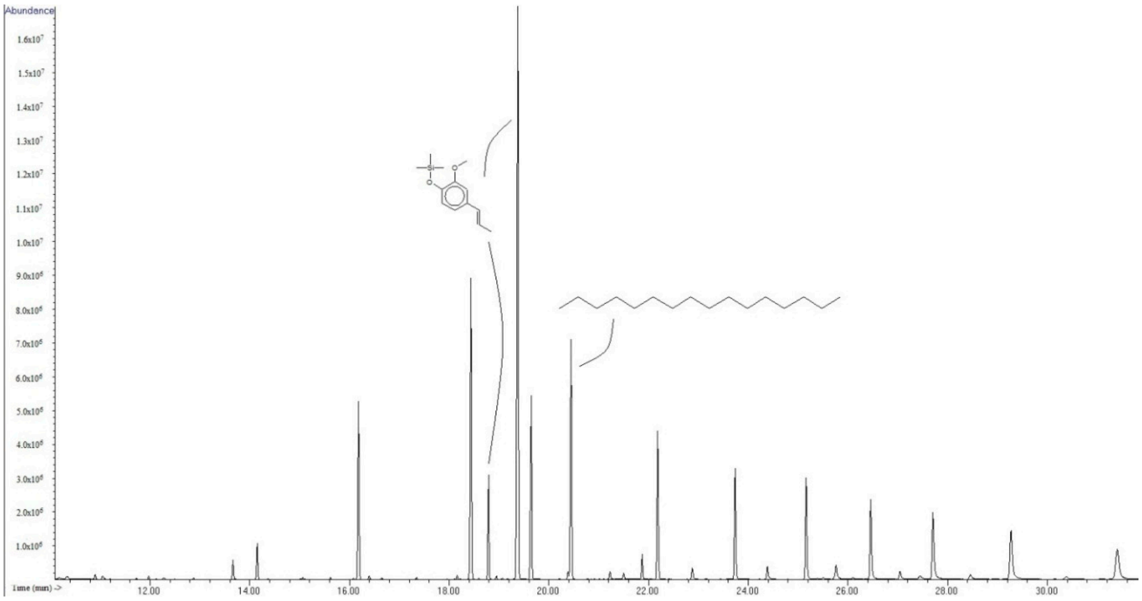


**Figure S1.** GC-MS chromatogram of a silylated sample of isoeugenol (mix of *cis* and *trans*) together with hexadecane which has reacted with the septum in the vial cap. All the unmarked peaks represent various silicon oxides.

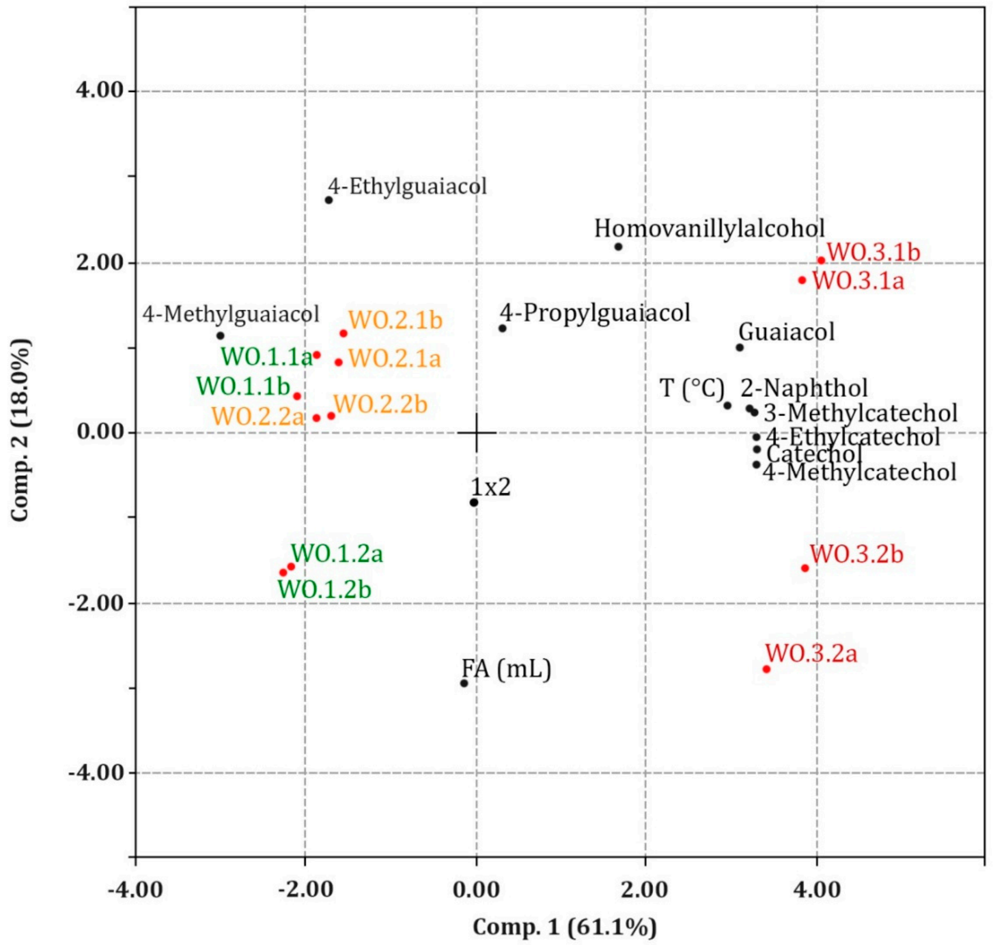


**Figure S2.** Biplot from principal component analysis (PCA) of data from LtL-oil quantification responses together with experimental coding. Colour coding of the experiments corresponds to the Van Krevelen plot in Figure 3 and the biplot in Figure 4 in the main text.

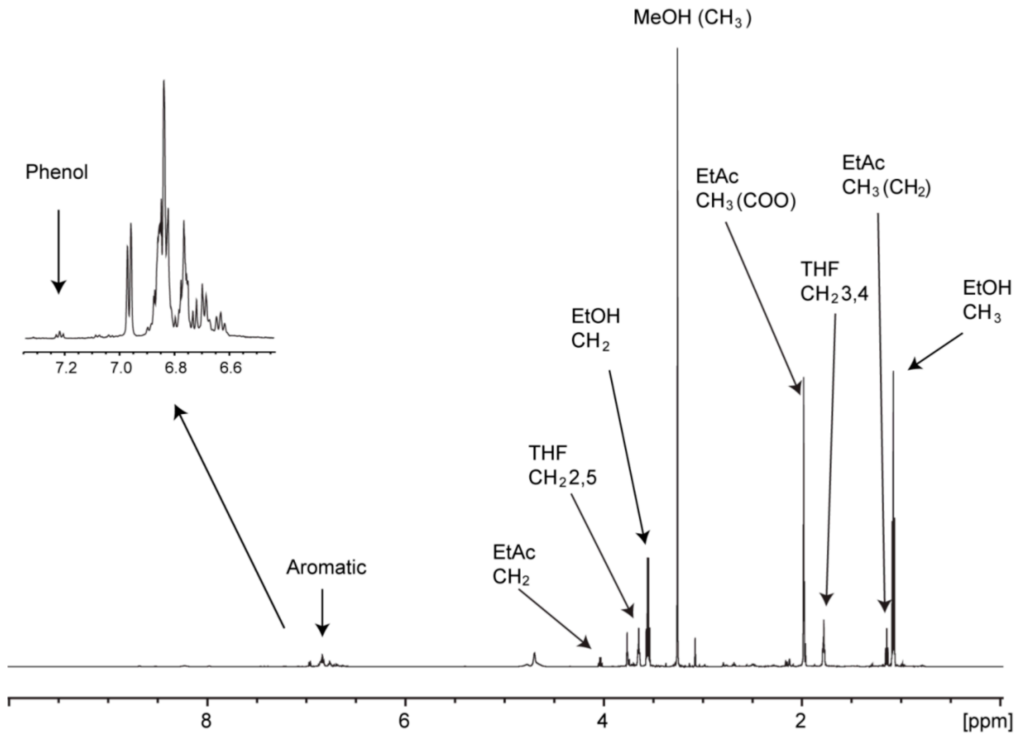


**Figure S3.** $^{1}$H-spectra of the aqueous phase from experiment WO.2.1.

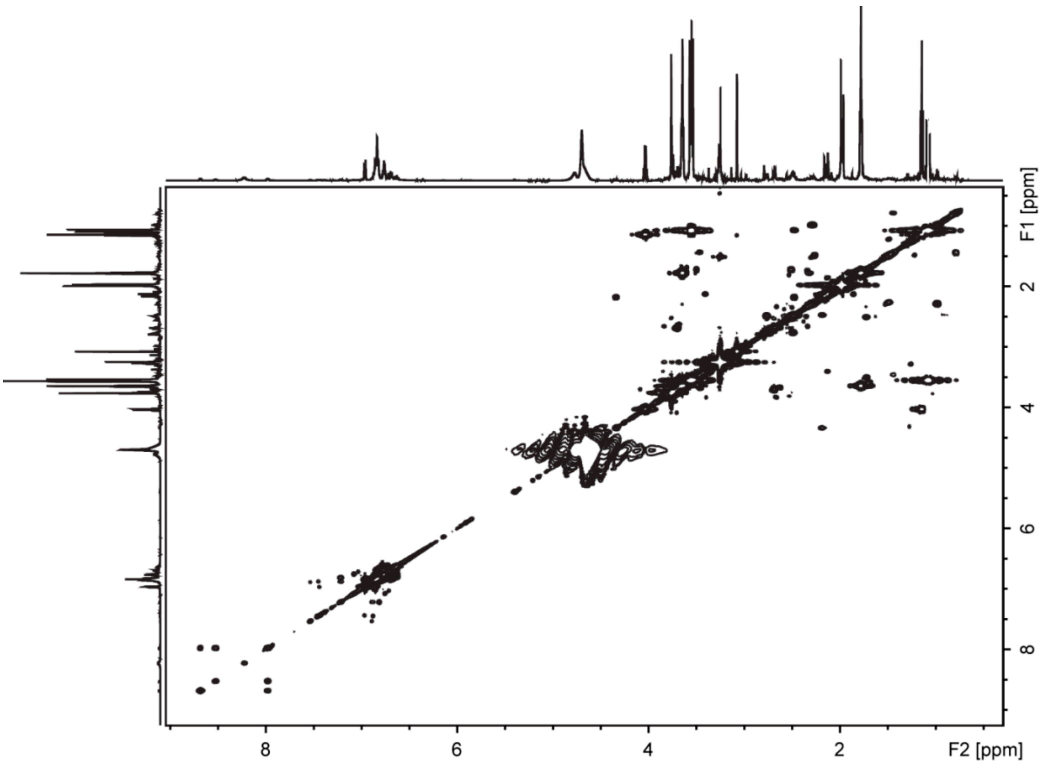


**Figure S4.** $^1H$-$^1H$-COSY spectra of the aqueous phase from experiment WO.2.1.

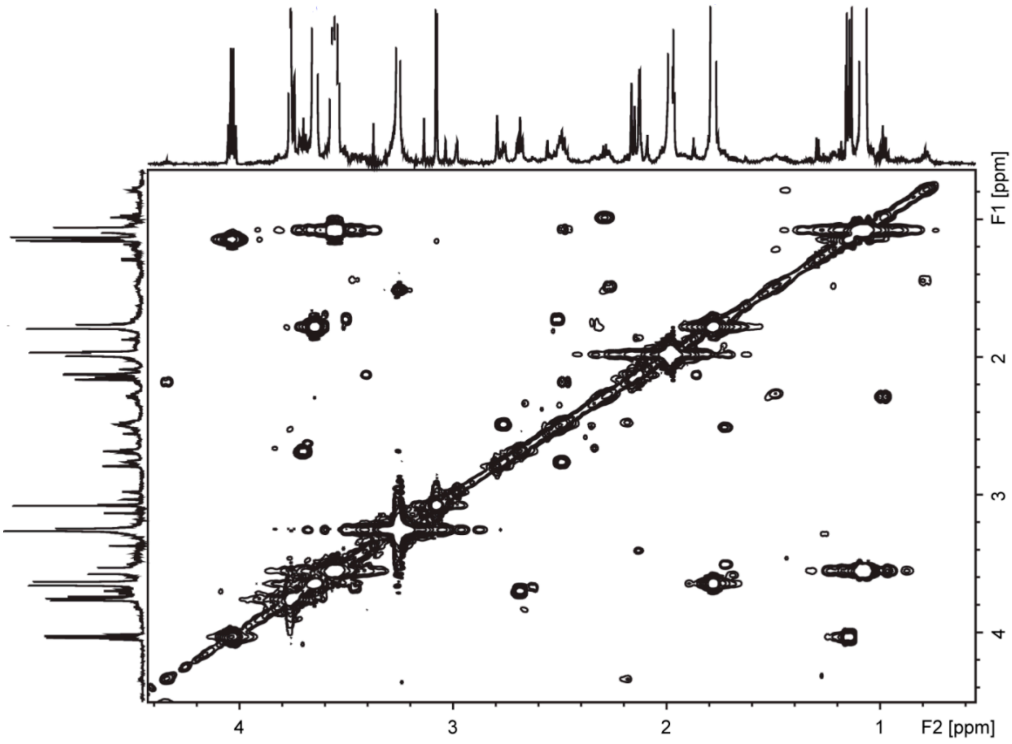


**Figure S5.** Enhanced $^1H$-$^1H$-COSY spectra of the aqueous phase from experiment WO.2.1 (4.4–0.6 ppm).

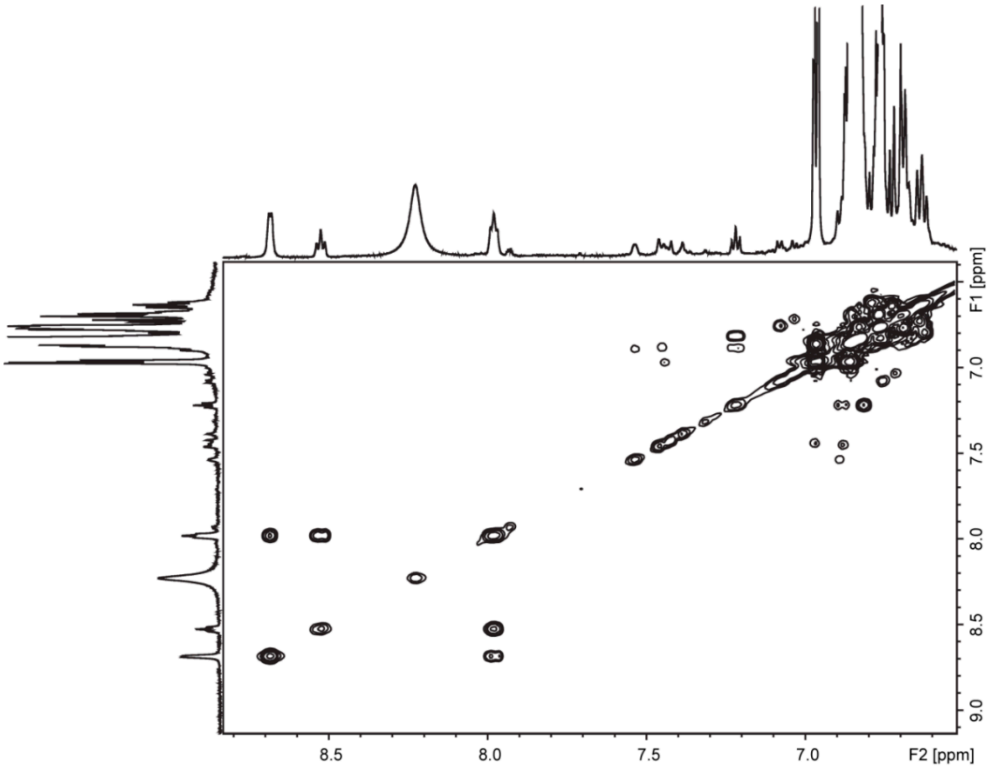


**Figure S6.** Enhanced $^1H$-$^1H$-COSY spectra of the aqueous phase from experiment WO.2.1 (8.8–6.5 ppm).
